# Sur8/Shoc2 promotes cell motility and metastasis through activation of Ras-PI3K signaling

**DOI:** 10.18632/oncotarget.5173

**Published:** 2015-09-05

**Authors:** Saluja Kaduwal, Woo-Jeong Jeong, Jong-Chan Park, Kug Hwa Lee, Young-Mi Lee, Soung-Hoo Jeon, Yong-Beom Lim, Do Sik Min, Kang-Yell Choi

**Affiliations:** ^1^ Translational Research Center for Protein Function Control, Yonsei University, Seoul, 120-749, Korea; ^2^ Department of Biotechnology, College of Life Science and Biotechnology, Yonsei University, Seoul, 120-749, Korea; ^3^ Department of Materials Science and Engineering, Yonsei University, Seoul, 120-749, Korea; ^4^ Department of Molecular Biology, College of Natural Science, Pusan National University, Pusan, 609-735, Korea; ^5^ Current address: Division of Pharmacology and Translational Research, Hanmi Research Center, Hwaseong-si Gyeonggi-do, 445-813, Korea; ^6^ Current address: Department of Microbiology and Immunology, Xenotransplantation Research Center, Medical Research Center, Institute of Endemic Disease, Seoul National University College of Medicine, Seoul, 110-799, Korea

**Keywords:** Sur8, Ras, PI3K, cell migration, metastasis

## Abstract

Sur8 (also known as Shoc2) is a Ras-Raf scaffold protein that modulates signaling through extracellular signal-regulated kinase (ERK) pathway. Although Sur8 has been shown to be a scaffold protein of the Ras-ERK pathway, its interaction with other signaling pathways and its involvement in tumor malignancy has not been reported. We identified that Sur8 interacts with the p110α subunit of phosphatidylinositol 3-kinase (PI3K), as well as with Ras and Raf, and these interactions are increased in an epidermal growth factor (EGF)- and oncogenic Ras-dependent manner. Sur8 regulates cell migration and invasion via activation of Rac and matrix metalloproteinases (MMPs). Interestingly, using inhibitors of MEK and PI3K we found Sur8 mediates these cellular behaviors predominantly through PI3K pathway. We further found that human metastatic melanoma tissues had higher Sur8 content followed by activations of Akt, ERK, and Rac. Lentivirus-mediated Sur8-knockdown attenuated metastatic potential of highly invasive B16-F10 melanoma cells indicating the role of Sur8 in melanoma metastasis. This is the first report to identify the role of scaffold protein Sur8 in regulating cell motility, invasion, and metastasis through activation of both ERK and PI3K pathways.

## INTRODUCTION

Ras proteins are small GTPases that cycle between inactive guanosine diphosphate (GDP)-bound and active guanosine triphosphate (GTP)-bound conformations. The exchange of GDP to GTP induces a conformational change that allows Ras to interact with various effector molecules, such as Raf and phosphatidylinositol 3-kinase (PI3K), and regulate various cellular responses including proliferation, survival, differentiation, motility, and tumorigenesis [1, 2]. The Raf-MEK-extracellular signal-regulated kinase (ERK) cascade is among the most, well-characterized signaling pathway that mediates Ras signaling. The activated Ras-Raf-ERK cascade is implicated in the activation of various target genes controlling cellular proliferation, differentiation, and transformation [3], as well as cell motility [4, 5]. Ras also activates the PI3K-Akt pathway by binding to the p110α catalytic subunit of class-I PI3Ks, which activates downstream kinases that affect cell growth and survival [6]. Activation of the PI3K pathway by oncogenic Ras is also implicated in cellular transformation and actin cytoskeleton rearrangement mediated by the small GTPase Rac [7], and binding of Ras to p110α is required for Ras-driven tumorigenesis in mice [8].

Organization of the several signaling pathways by Ras is strictly coordinated by the scaffold proteins [9], which shield correct signaling proteins from irrelevant stimuli by coordinating positive and negative signals, providing signal specificity, and modulating signal intensity [10–12].

Sur8 (also known as Shoc2) is a scaffold protein that positively regulates Ras-mediated signal transduction during *C. elegans* vulval development [13]. The human homolog of Sur8 is a conserved leucine-repeat rich protein involved in fibroblast growth factor receptor signaling [14]. Sur8 is reported to interact with H-, K-, N-Ras and enhance the ability of all these Ras isoforms to activate ERK [13, 15]. However, other studies have reported Sur8 interacts only with M-Ras but not with other isoforms of Ras to regulate ERK pathway [16, 17]. Although Sur8 has been reported as a positive regulator of Ras-ERK pathway, its interaction with other signaling pathways and its involvement in pathophysiological conditions is mostly unknown.

Here, we show for the first time that Sur8 interacts not only with Ras and Raf but also with p110α subunit of PI3K and these interactions are important in Sur8-mediated cell migration and invasion, along with tumor metastasis. Mechanistically, Sur8-regulated these pathophysiologies through activation of Rac and matrix metalloproteinases (MMPs) predominantly through the PI3K pathway. Our study provides a novel paradigm for scaffold protein Sur8 as a positive regulator of tumor malignancy through the Ras-PI3K-Rac-MMP signaling and a potential novel therapeutic target for suppressing tumor metastasis that arises from Ras/PI3K-induced activations of both the Raf and Akt pathways.

## RESULTS

### Sur8 plays a role in cell migration

**Figure 1:**
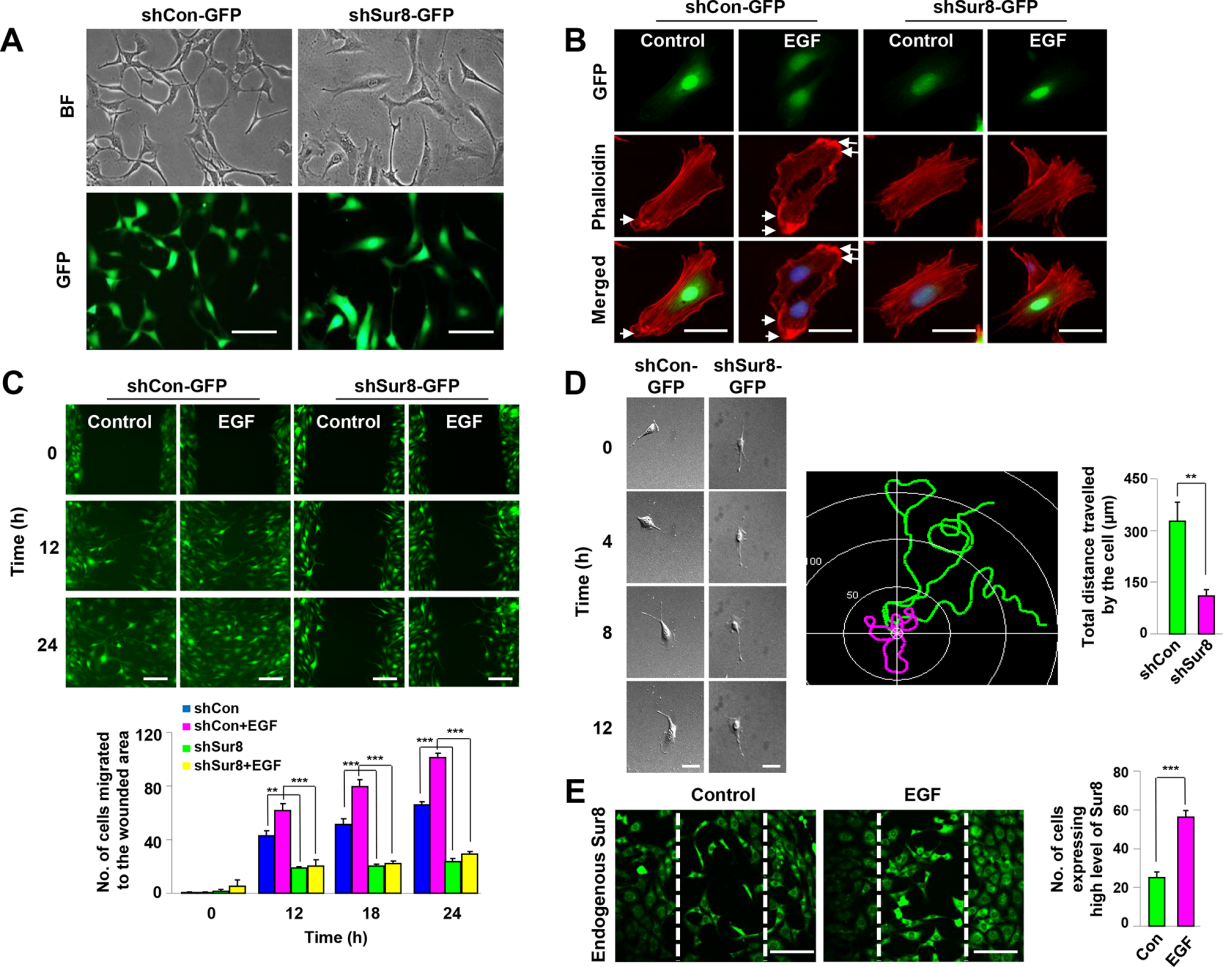
Role of Sur8 in actin cytoskeleton rearrangement and cell migration The shCon-GFP and shSur8-GFP NIH3T3 cells are presented in **A.** Cells were grown on DMEM media. Representative bright and GFP field images showing the cell morphology after 48 hours of seeding were captured using a Nikon TE-2000U microscope. Scale bars, 250 μm. **B.** Cells treated with EGF for 24 hours were stained with phalloidin red and counterstained with DAPI. Arrowheads indicate lamellipodia. Scale bars, 50 μm. **C.** Confluent cells were scratched, and treated with EGF. Cell migratory behavior was assessed using real-time imaging. Scale bars, 250 μm. Values are mean ± s.e.m. of three independent experiments. **D.** Single-cell migratory behavior was monitored using real-time imaging for at least three independent times, values are mean ± s.e.m. Scale bars, 100 μm. **E.** Confluent NIH3T3 cells were scratched and either treated or non-treated with EGF for 15 hours. Immunocytochemistry was performed using an anti-Sur8 antibody and the experiment was performed for three independent times, values are mean ± s.e.m. Scale bars, 250 μm.

Although the involvement of Ras signaling in the regulation of actin rearrangement and cell motility is reported [5, 7], the role of Sur8 in these processes has not been characterized. Because Sur8 regulates Ras signaling, we aimed to determine the role of Sur8 in cell migration by generating a Sur8 knocked down stable NIH3T3 cell line using a green fluorescent protein (GFP)-tagged lentivirus. Stable knockdown of Sur8 in NIH3T3 cells (shSur8-GFP) decreased epidermal growth factor (EGF)-induced activation of ERKs and Elk-1 reporter compared to control (shCon-GFP) cells (Supplementary Figure 1A and 1B). The shSur8-GFP NIH3T3 cells had a flatter morphology with pointed protrusions on the ends (Figure 1A), whereas the shCon-GFP cells were extended and elongated with a typical fibroblast phenotype [18].

Because changes in the cell morphology is associated with actin cytoskeletal rearrangement [19], we performed actin staining in shCon-GFP and shSur8-GFP NIH3T3 cells with or without EGF treatment (Figure 1B). EGF-treated shCon-GFP cells formed concentrated actin bundles around the cell tip representing lamellipodia of a migrating cell [19, 20], whereas shSur8-GFP cells did not (Figure 1B). The red fluorescent protein (RFP)-tagged actin (RFP-actin) also failed to localize around the cell periphery in shSur8-GFP NIH3T3 cells (Supplementary Figure 1C).

Because actin rearrangement is involved in cell migration, we monitored the wound healing capacities of shCon-GFP and shSur8-GFP NIH3T3 cells using real-time imaging. Sur8 knockdown decreased the migratory capacity of cells even in EGF-treated conditions compared to shCon-GFP cells (Figure 1C). Sur8 knockdown also decreased ERKs phosphorylation and cell migration in oncogenic H-Ras-overexpressing NIH3T3 cells (Supplementary Figure 1D–1F).

Single cell migration of shCon-GFP and shSur8-GFP NIH3T3 cells captured by real-time imaging confirmed that Sur8 knockdown reduced the ability of each cell to migrate (Figure 1D). In the wound-healing assay, in both EGF treated or non-treated conditions, the cells migrating towards the center to heal the wound expressed higher levels of endogenous Sur8 protein compared to the cells on the boundary of the wound, further highlighting the importance of Sur8 role in cell migration (Figure 1E).

### Sur8 regulates EGF- and oncogenic H-Ras-mediated activation of Rac and Akt

**Figure 2:**
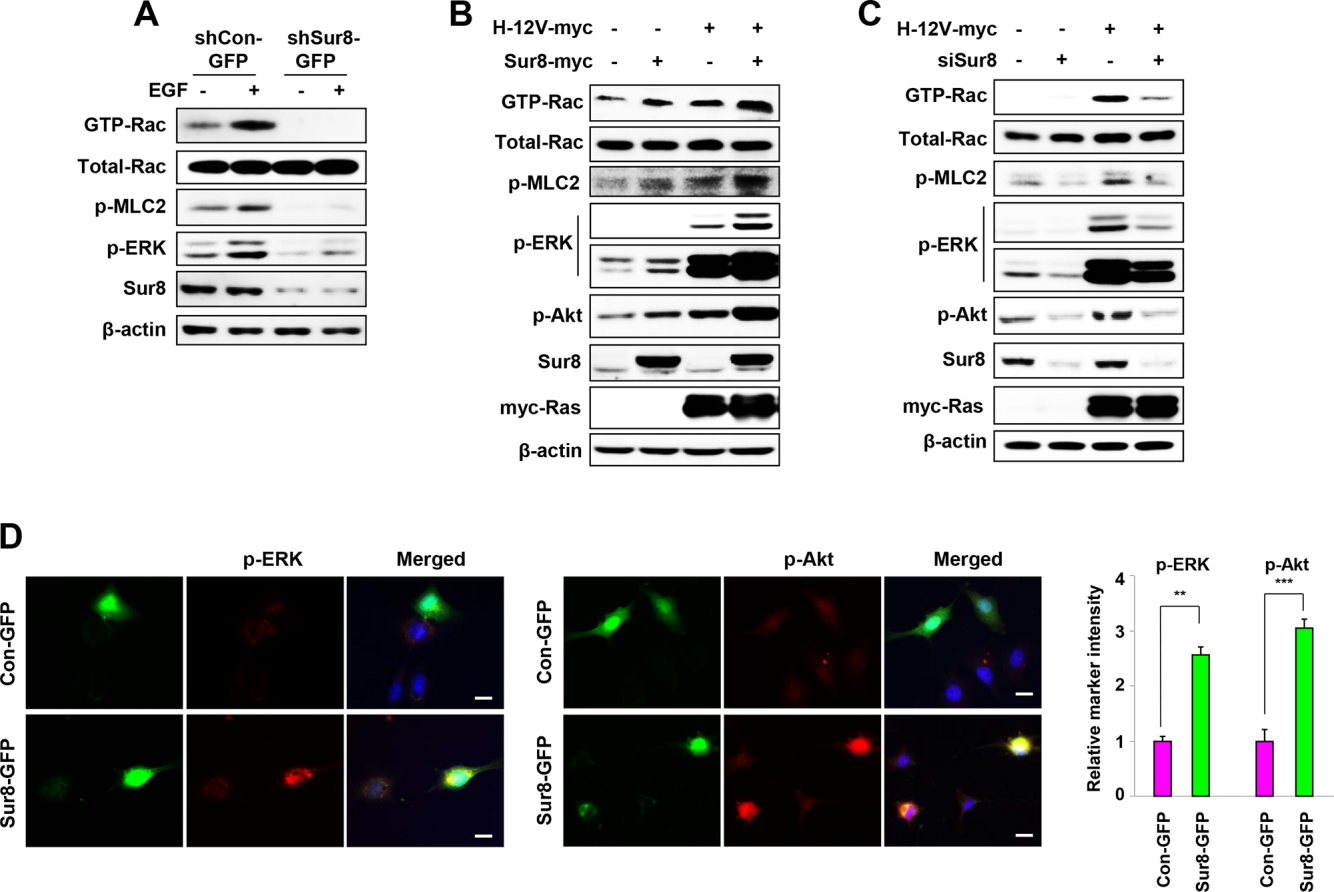
Effect of Sur8 on Rac and Akt activation **A–C.** The shCon-GFP and shSur8-GFP NIH3T3 cells were treated with EGF for 10 minutes (A), HEK293 cells were transfected with the indicated plasmids or siRNAs (siGFP or siSur8 #1 and #2) (B, C). For GTP-Rac measurement, WCLs were incubated with GST-PAK-CD and analyzed by immunoblotting with an anti-Rac1 antibody. For all other measurements, WCLs were immunoblotted against the indicated proteins. **D.** NIH3T3 cells were transfected with either Con-GFP or Sur8-GFP plasmids, and immunocytochemical analysis was performed using anti-p-ERK or -p-Akt antibody. Cell nuclei were counterstained with DAPI. Relative intensities of the markers stained were quantified for at least 15 different cells using NIS-Elements AR 3.1. Scale bars, 50 μm. The values are mean ± s.e.m. of three independent experiments.

Rac-GTPases are involved in actin rearrangement, which leads to altered cell morphology and increased cell migration [21]. Therefore, we examined whether Sur8 regulated Rac activation. The GTP-Rac level was undetectable in shSur8-GFP NIH3T3 cells in both normal and EGF-treated conditions, indicating these cells were unable to activate Rac through EGF signaling (Figure 2A). A positive association between Rac activation and phosphorylation of MLC2, a downstream effector of activated Rac [22] was also observed (Figure 2A). Similarly, Sur8 overexpression in human embryonic kidney (HEK) 293 cells increased Rac activation (Figure 2B), and Sur8 overexpression together with oncogenic H-Ras further enhanced oncogenic H-Ras-mediated Rac activation. The phosphorylation of ERKs and MLC2 were increased by Sur8 overexpression, and coexpression with oncogenic H-Ras further enhanced ERKs and MLC2 phosphorylation (Figure 2B). Interestingly, we also observed the enhanced Akt phosphorylation by Sur8 overexpression and the presence of Sur8 further enhanced the oncogenic H-Ras-mediated Akt phosphorylation (Figure 2B). Conversely, Sur8 knockdown decreased Rac activation and phosphorylation of ERKs, MLC2, and Akt even in the presence of oncogenic H-Ras (Figure 2C). The Sur8-mediated effects on ERKs and Akt phosphorylation were further clarified by performing immunocytochemistry analyses of NIH3T3 cells overexpressing GFP-tagged Sur8 (Sur8-GFP) (Figure 2D).

### Sur8 mediates cell migration through Ras-PI3K-Rac pathway

**Figure 3:**
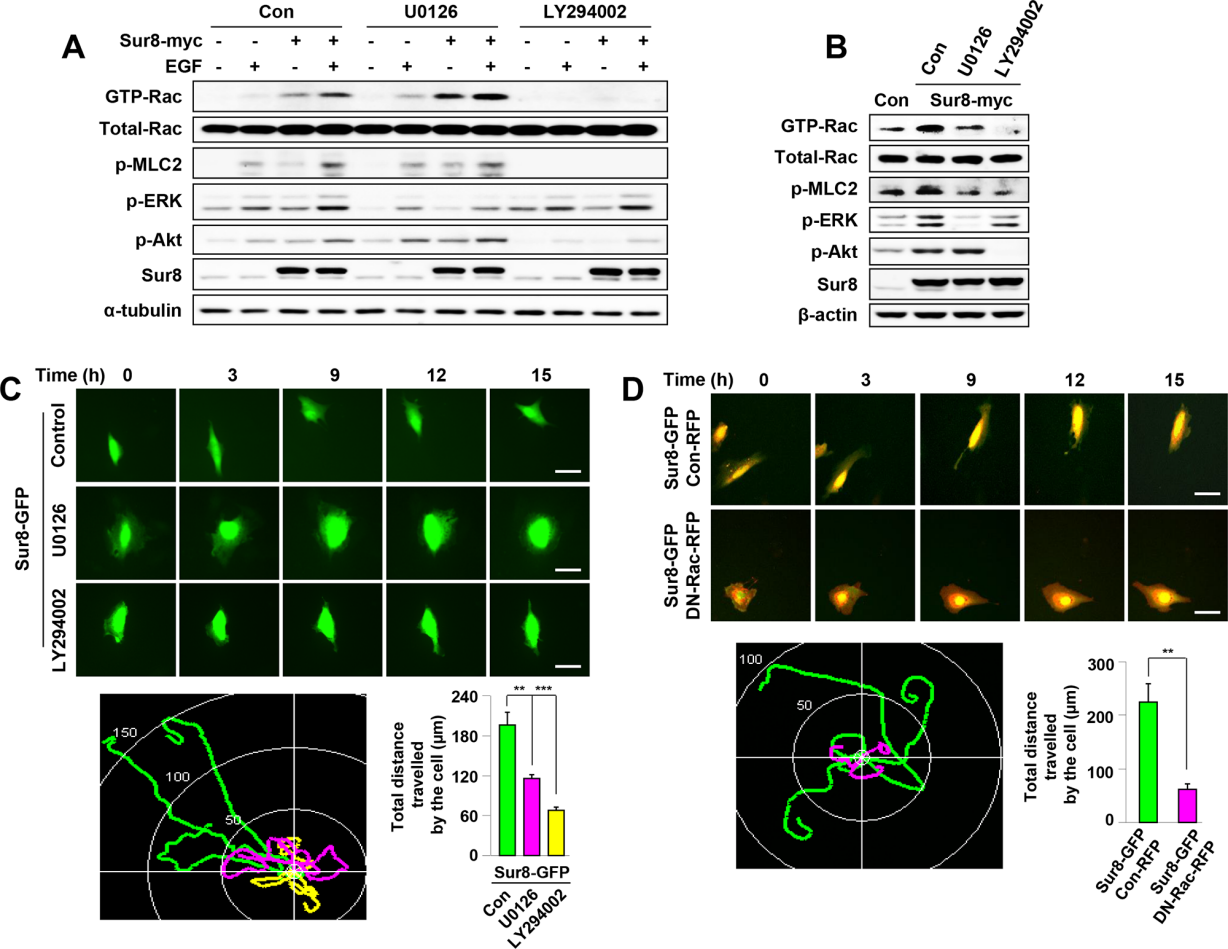
Effects of Ras-ERK or PI3K pathway inhibition on Sur8-mediated Rac activation and cell migration **A, B.** WCLs of HEK293 cells transfected with the indicated plasmids and treated with either U0126 or LY294002 for 1 hour (A) or 15 hours (B) were incubated with GST-PAK-CD. GTP-Rac was measured as described in Figure 2. WCL aliquots were immunoblotted using the indicated antibodies. Cells in (A) were treated with EGF for 10 minutes. **C.** NIH3T3 cells were transfected with Sur8-GFP and treated with U0126 or LY294002. Real-time imaging was performed to monitor cell migration and the migratory paths were recorded and quantified from three independent experiments using NIS-Elements AR 3.1, values are mean ± s.e.m. Scale bars, 50 μm. **D.** NIH3T3 cells co-expressing Sur8-GFP and Con-RFP; or Sur8-GFP and DN-Rac-RFP were imaged to monitor cell migration and the migratory paths were recorded and quantified from three independent experiments using NIS-Elements AR 3.1, values are mean ± s.e.m. Scale bars, 50 μm.

Previous studies have illustrated the involvement of Ras-ERK and Ras-PI3K pathways in regulating Rac activation [23, 24]. To understand molecular mechanisms underlying Sur8-mediated Rac activation, we examined the effect of MEK or PI3K inhibitor on Sur8-mediated Rac activation. As shown by immunoblot analyses, inhibiting ERK activity with the MEK inhibitor U0126 for 1 hour did not reduce Sur8-mediated activation of Rac and MLC2 (Figure 3A), but 15 hours U0126 treatment partially reduced Sur8-mediated activation of Rac and MLC2 (Figure 3B). However, incubation with the PI3K inhibitor LY294002 for both 1 hour and 15 hours abolished Sur8-mediated Rac activation (Figure 3A and 3B).

Real-time imaging analyses showed that NIH3T3 cells overexpressing Sur8-GFP migrated faster than the control-GFP (Con-GFP)-expressing cells (Supplementary Figure 2A). Cell migration was more strongly inhibited by LY294002 than by U0126 in Sur8-GFP overexpressing NIH3T3 cells (Figure 3C). Furthermore, the migration of Sur8-GFP overexpressing NIH3T3 cells was attenuated by the overexpression of RFP-tagged dominant-negative Rac (DN-Rac-RFP) (Figure 3D) and transient overexpression of RFP-tagged constitutively active Rac (CA-Rac-RFP) in shSur8-GFP NIH3T3 cells partially rescued cell migration (Supplementary Figure 3A and 3B). Collectively, Sur8 mediates cell migration primarily through the PI3K pathway mediated Rac activation, although the ERK pathway also plays a minor role.

### Sur8 regulates EGF- and oncogenic H-Ras-induced invasive properties of cells

**Figure 4:**
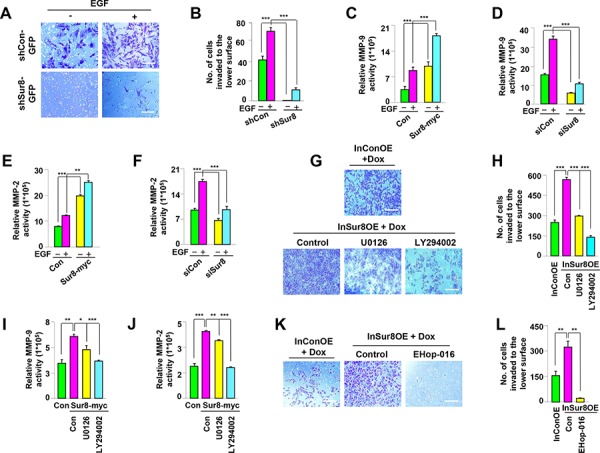
Role of Sur8 in regulating MMP activity and cell invasion **A, B.** The shCon-GFP and shSur8-GFP NIH3T3 cells were seeded on matrigel-coated chambers. EGF was treated on the lower surface. Cells that invaded through the matrigel chambers were stained with crystal violet. Representative images were captured, and the cells were counted from three independent experiments. Scale bars, 250 μm. **C–F.** Activation of the MMP-9 and MMP-2 promoters in response to Sur8 overexpression (C, E), and knockdown (D, F), in untreated or EGF-treated HEK293 cells were measured as described in Materials and methods. **G, H.** InConOE and InSur8OE NIH3T3 cells were treated with Dox and seeded on matrigel-coated chambers. U0126 or LY294002 was treated on the lower surface. The cells that invaded through the matrigel were stained with crystal violet. Representative images were captured, and the total number of invaded cells is presented. Experiment was performed for three independent times. Scale bars, 200 μm. **I, J.** Sur8-induced activation of the MMP-9 and MMP-2 promoters in response to U0126 or LY294002 in HEK293 cells were measured as described in Materials and methods. **K, L.** InConOE and InSur8OE NIH3T3 cells were treated with Dox and seeded on matrigel-coated chambers. EHop-016 was treated on the lower surface. The cells that invaded through the matrigel were stained with crystal violet. Representative images were captured, and the total number of invaded cells is presented. Experiment was confirmed for three independent times. Scale bars, 200 μm. InConOE and InSur8OE NIH3T3 cells were treated with Dox for 72 hours before the cells were seeded. All the values in figures are mean ± s.e.m.

To establish whether Sur8 plays a role in cell invasion, matrigel invasion assays were performed. Sur8 knockdown resulted in the inability of NIH3T3 cells to pass through the matrigel, and this effect was not reversed even with EGF treatment (Figure 4A and 4B). Similarly, oncogenic H-Ras-induced cell invasion was also attenuated by Sur8 knockdown (Supplementary Figure 4A and 4B). Ras signaling activates MMPs [25, 26], which regulate the extracellular matrix (ECM) degradation that enhances cell invasion [27]. Therefore, we investigated whether Sur8-mediated cell invasion is executed through the regulation of MMP-9 and MMP-2. The EGF-induced promoter activities of MMP-9 and MMP-2 were increased and decreased by Sur8 overexpression (Figure 4C and 4E) and knockdown (Figure 4D and 4F), respectively. Similarly, increased activities of MMP-9 and MMP-2 promoters induced by oncogenic H-Ras overexpression were further enhanced and attenuated by ectopic expression (Supplementary Figure 4C and 4D) and knockdown (Supplementary Figure 4E and 4F) of Sur8, respectively. The doxycycline (Dox)-inducible Sur8-overexpressing NIH3T3 cells (InSur8OE) showed increased phosphorylation of Akt and ERKs, as well as enhanced wound healing compared to the control cells (InConOE; Supplementary Figure 5A–5C). Consistent with the important role of PI3K signaling in Sur8-mediated cell migration (Figure 3C), inhibition of PI3K reduced Sur8-mediated cell invasion (Figure 4G and 4H), and MMP-9 and MMP-2 promoter activities (Figure 4I and 4J), more than inhibition of MEK. Similar to the results obtained using U0126 or LY294002 (Figure 3C, 4G and 4H), the more significant role of the PI3K pathway compared to the ERK pathway in the Sur8-mediated migration and invasion of cells was also confirmed by using other MEK and PI3K specific inhibitors (AS703026 and GDC0941, respectively), (Supplementary Figure 6A–6D). Furthermore, treatment of the GTP-Rac inhibitor EHOp-016 significantly inhibited the Sur8 overexpression-mediated invasive properties of NIH3T3 cells (Figure 4K and 4L) to a level comparable to the dual inhibition of ERK and PI3K pathways (Supplementary Figure 7A and 7B). In addition, transient overexpression of CA-Rac-RFP in shSur8-GFP NIH3T3 cells partially rescued invasion properties (Supplementary Figure 7C and 7D). Overall, both ERK and PI3K pathways are likely involved in Sur8-mediated Rac activation and MMP signaling even though PI3K plays a major role.

### Sur8 forms a complex with Ras, Raf, and the p110α subunit of PI3K, and the interaction is increased in an EGF- and oncogenic Ras-dependent manner

**Figure 5:**
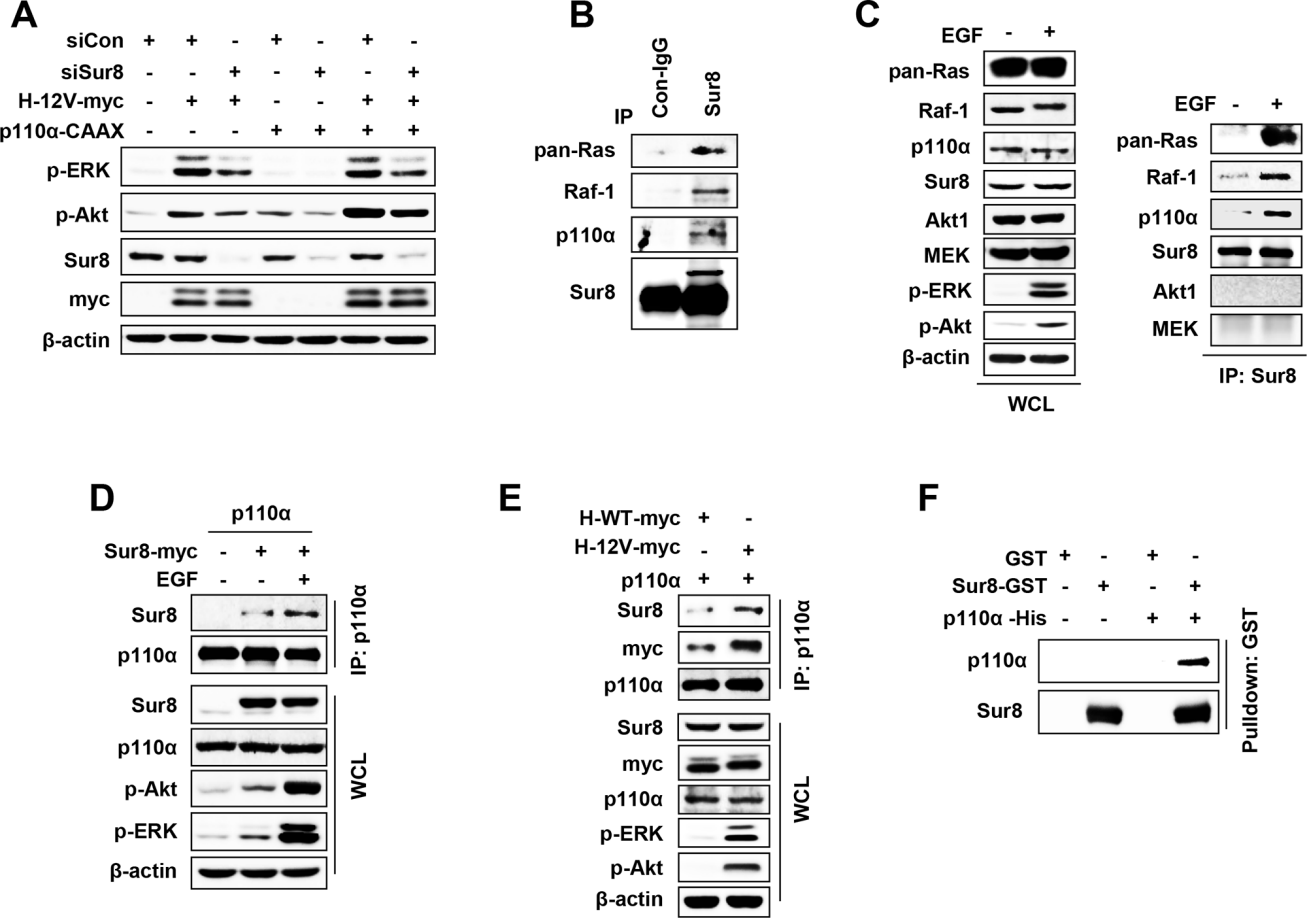
Sur8 interacts with the p110α subunit of PI3K in an EGF- and oncogenic Ras-dependent manner **A.** HEK293 cells were transfected with the indicated plasmids or siRNAs (siGFP or siSur8 #1 and #2), and WCLs were immunoblotted against the indicated proteins. **B.** WCLs of HEK293 cells were immunoprecipitated with either control IgG or Sur8 antibody and immunoblotting was performed against the indicated proteins. **C–E.** HEK293 cells were transfected with the indicated plasmids, treated or non-treated with EGF and WCLs were immunoprecipitated with either anti-Sur8 (C) or -p110α (D, E) antibody and immunoblottings were performed against the indicated proteins. **F.** GST and GST-Sur8 were incubated with His-p110α recombinant protein (3 μg each) for 1 hour at 4°C. Glutathione agarose beads were used to pull down p110α as described in Materials and methods. Immunoblotting was performed using anti-Sur8 or -p110α antibody. Cells in figures (C) and (D) were treated with EGF for 10 minutes.

Because we observed that Sur8 regulated both Ras-ERK and PI3K signaling, we sought to investigate how Sur8 regulates Ras-PI3K signaling. Sur8 knockdown attenuated both oncogenic H-Ras-mediated phosphorylation of ERKs and Akt and active p110α-induced phosphorylation of Akt (Figure 5A).

Because we observed that the migratory and invasive properties of cells by Sur8 were executed through the PI3K signaling, we investigated whether Sur8 can bind to PI3K-Akt pathway components. Immunoprecipitation (IP) assays performed in HEK293 cells revealed that Sur8 binds to not only Ras and Raf-1 but also to p110α at the endogenous protein level (Figure 5B) and these bindings were further enhanced by EGF treatment (Figure 5C). However, Sur8 did not bind with Akt or MEK (Figure 5C) (downstream effectors of p110α and Raf-1, respectively), even in EGF-treated cells. Reverse IP confirmed that p110α binds to Sur8, and EGF treatment further increased this binding affinity (Figure 5D). Similar to EGF, oncogenic H-Ras also increased the complex formation between p110α and Sur8 more than wild type H-Ras in HEK293 cells (Figure 5E).

Because Sur8 formed a complex with Ras, Raf-1, and p110α, respectively, and regulated both the Ras-ERK and PI3K pathways, we sought to investigate whether Sur8 directly interacts with p110α. An *in vitro* binding assay using recombinant GST-Sur8 and His-p110α proteins showed a direct interaction between Sur8 and p110α (Figure 5F).

### Sur8 knockdown reduces phosphorylation of ERKs and Akt, cell migration, invasion, and lung metastasis of B16-F10 melanoma cells

**Figure 6:**
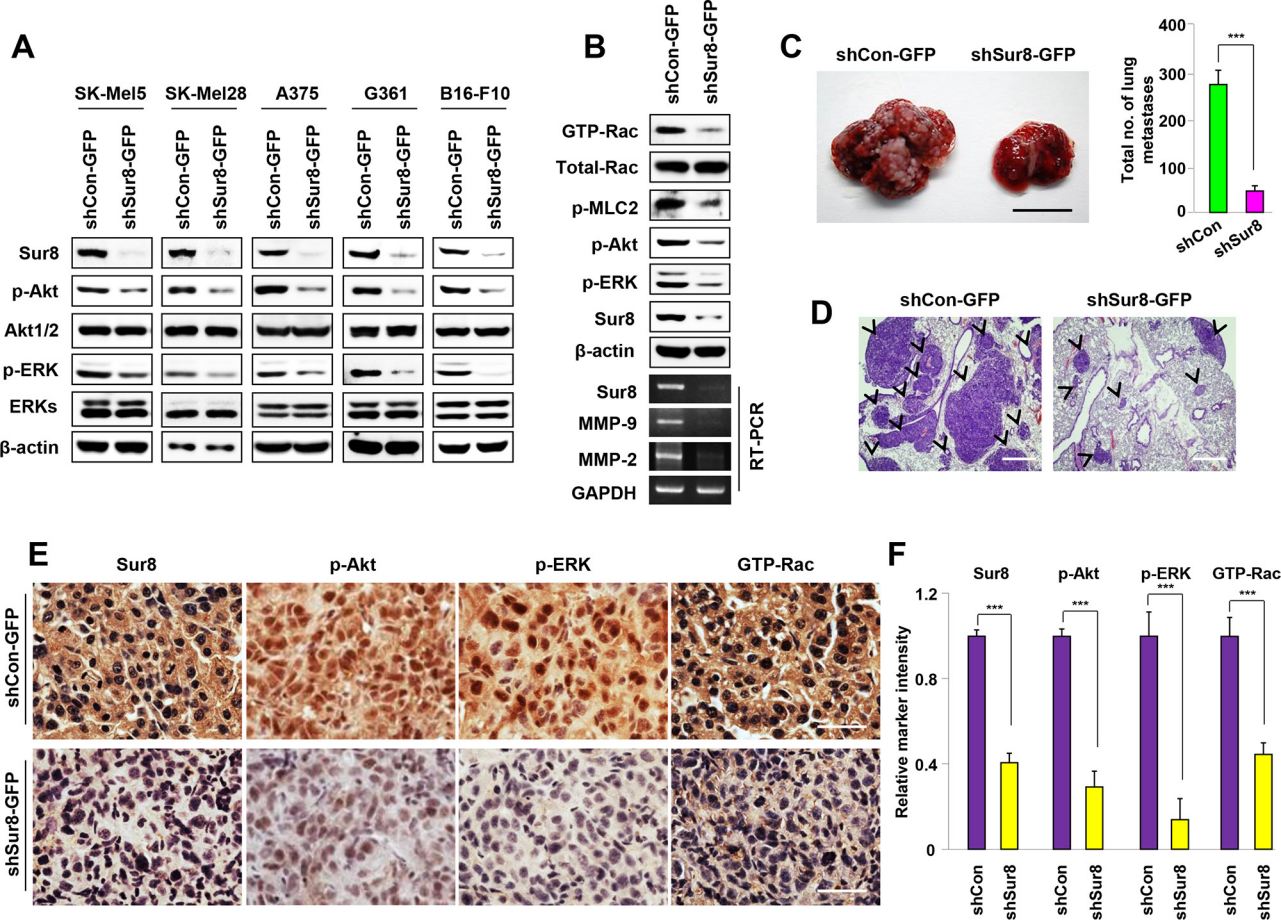
Effects of Sur8 knockdown on ERKs and Akt phosphorylation, Rac activation, MMPs expression, and lung metastasis in B16-F10 melanoma cells **A.** SK-Mel5, SK-Mel28, A375, and G361 cells were infected with Sur8-knockdown lentiviruses (shSur8 #1 and #2) for 72 hours. The WCLs of these cells and B16-F10 melanoma cells with stable Sur8 knockdown were immunoblotted against the indicated proteins. **B.** shCon-GFP and shSur8-GFP B16-F10 cells were used. WCLs were incubated with GST-PAK-CD, GTP-Rac was detected with an anti-Rac1 antibody, other indicated proteins were detected by immunoblotting with specific antibodies, and RT-PCR analyses of Sur8, MMP-9, MMP-2, and GAPDH were performed as described in Materials and methods. **C–F.** The shCon-GFP (*n* = 6) and shSur8-GFP (*n* = 8) B16-F10 cells were injected into the tail vein of C57BL/6 mice. Lung metastases were observed at 21 days. (C) Representative gross images and quantification of lung metastases are presented. Scale bar, 1 cm. (D) Paraffin-embedded metastasized lung tissue sections were subjected to H&E staining, scale bars, 200 μm; or (E, F) DAB analyses using anti-Sur8, -p-Akt, -p-ERK, or -GTP-Rac antibody, scale bars, 25 μm. Arrowheads indicate lung tumor. Relative marker intensities were quantified in (F) using HistoQuest software for at least 5 different samples in each experimental case. Values in figures (C) and (F) are calculated using student's *t* test.

Melanoma is a highly metastatic skin cancer and is the most frequent cause of mortality attributed to metastasis. Several studies have demonstrated aberrant high activities of Ras and PI3K pathways in melanomas [28]. Based on these observations, we investigated whether Sur8 knockdown regulates the metastatic potential of melanomas through regulation of Ras/PI3K signaling. Immunoblotting analyses showed that activities of ERKs and Akt were significantly reduced by Sur8 knockdown in all of the melanoma cell lines tested (SK-Mel5, SK-Mel28, A375, G361, and B16-F10) (Figure 6A), and Sur8 interacted with p110α, Ras, and Raf-1 in B16-F10 cells at the endogenous protein level (Supplementary Figure 8A). Because B16-F10 cells are widely used *in vivo* as a model of metastasis because of their highly metastatic potential [29], we first confirmed the involvement of ERK, PI3K, and Rac pathways in B16-F10 cell migration by using specific inhibitors of these respective pathways (Supplementary Figure 8B). Furthermore, stable knockdown of Sur8 in B16-F10 cells significantly down regulated Rac activation and mRNA expressions of MMP-9 and MMP-2 (Figure 6B). Cell migratory potential and invasive properties were also strongly inhibited by stable knockdown of Sur8 in B16-F10 cells (Supplementary Figure 8C and 8D). Next, we performed an *in vivo* lung metastasis assay by injecting stable Sur8 knockdown B16-F10 cells into the tail vein of C57BL/6 mice. Mice injected with scrambled virus-infected cells had visible metastasized lung tumors after 3 weeks. However, mice injected with Sur8 knockdown B16-F10 cells had minimal metastasized lung tumors (Figure 6C), which was confirmed by hematoxylin and eosin (H&E) staining of the lung tissue sections (Figure 6D). Furthermore, mice injected with the lentivirus-mediated Sur8 knockdown cells had lower signals for Sur8, p-Akt, p-ERK, and GTP-Rac in their tumor tissues than those injected with scrambled virus-infected cells (Figure 6E and 6F). Collectively, Sur8 likely modulates the cancer cell motility, invasion, and metastatic potential by regulating Ras-PI3K-Rac-MMP signaling.

### Sur8 was overexpressed followed by the activation of Akt, ERKs, and Rac in human metastatic melanoma

**Figure 7:**
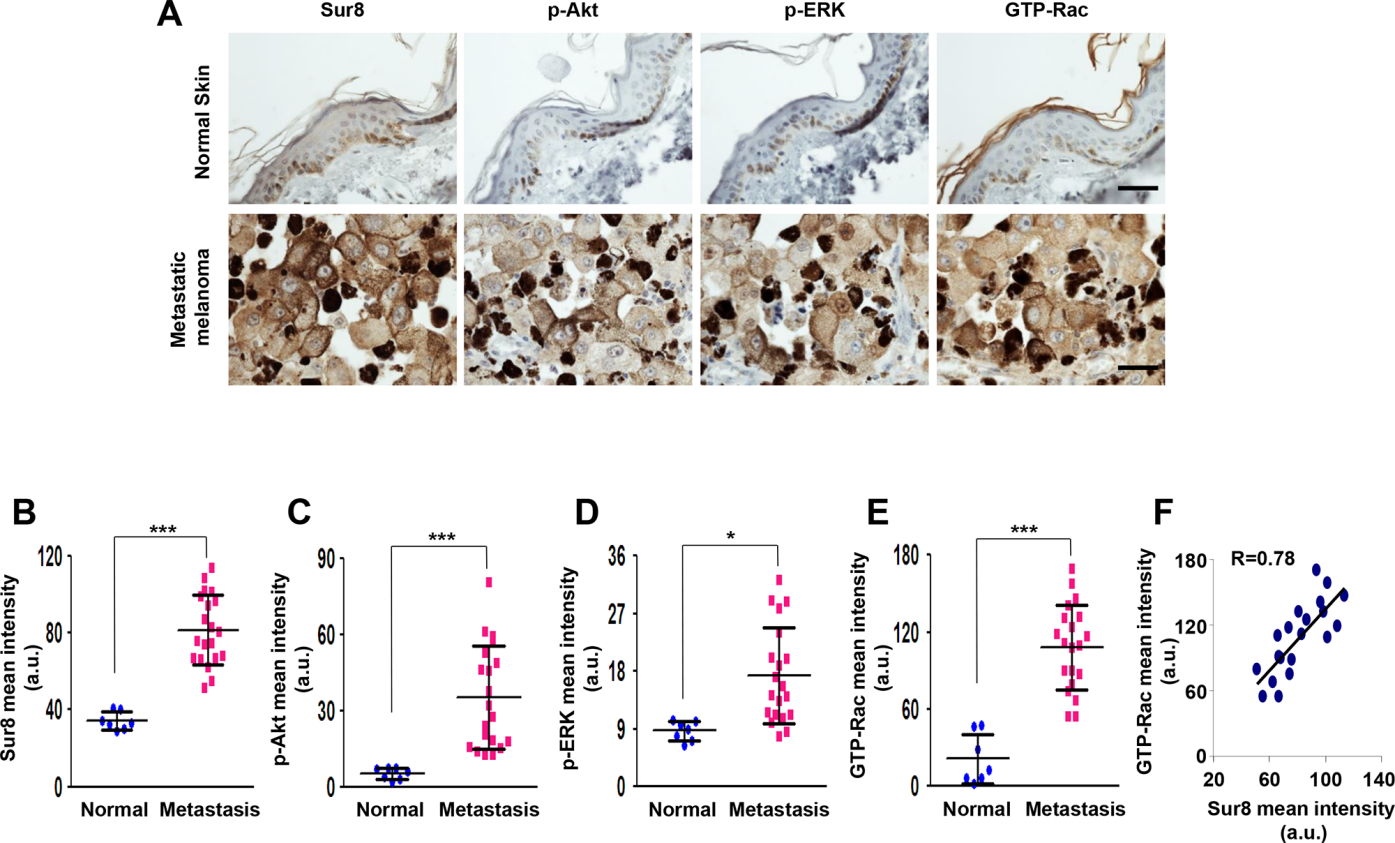
The expression of Sur8, p-Akt, p-ERK, and GTP-Rac in human metastatic melanoma **A–E.** Representative DAB staining analyses using anti-Sur8, -p-Akt, -p-ERK, or -GTP-Rac antibody in normal skin (*n* = 15) and metastatic melanoma tissues (*n* = 64) using a human melanoma TMA (A) The expressions were captured and quantified using HistoQuest software for at least 7 independent normal skin specimens and 20 independent metastatic melanoma specimens and represented as scattered dot plots (B-E) Scale bars, 25 μm. **F.** Graph showing the correlation between Sur8 and GTP-Rac expression. A Pearson Correlation Coefficient test was used to calculate the correlation coefficient (R) a.u., arbitrary units. All the values in figures are calculated using student's *t* test.

To validate the possible role of Sur8 in the metastasis of human melanoma and to confirm our *in vitro* findings showing an association between Sur8 and activation of Akt, ERKs, and Rac, we utilized a tissue microarray (TMA; ME2082b; US Biomax) consisting of human metastatic melanoma (*n* = 64) and normal skin tissue (*n* = 15) specimens. We quantified results of the immunohistochemistry (IHC) stainings using HistoQuest software (TissueGnostics, Vienna, Austria) and observed that Sur8, p-Akt, p-ERK, and GTP-Rac were highly expressed in the identical human metastatic melanoma tissues compared with normal skin tissues (Figure 7A–7E). Moreover, expression pattern of Sur8 highly correlated with expression of GTP-Rac in the identical metastatic melanoma specimens which were confirmed using quantitative analyses of IHC staining data (Figure 7F). Thus, these results indicate that Sur8 was upregulated during human melanoma metastasis and resulted in the concomitant increase of Akt and ERK phosphorylation leading to the activation of Rac.

## DISCUSSION

The current study is the first to reveal that Ras-Raf scaffold protein Sur8 plays an essential role in the regulation of not only ERK but also PI3K pathway and is involved in cell migration, invasion, and metastasis. The role of Sur8 in tumor malignancy was characterized *in vivo* by injecting highly metastatic B16-F10 cells into mice and using human metastatic melanoma samples and through various *in vitro* analyses.

Tumor metastasis is a multi-step process that requires the concerted action of several cellular processes, including increased cell motility, invasiveness, and survival signaling, that leads to the dissemination of the primary tumor to the distant site [30, 31]. In our study, Sur8 knockdown led to rearrangement of the actin cytoskeleton and the abolishment of lamellipodia-like structures at the leading edges of cells, and it was also associated with reduced single-cell migratory potential and wound healing capacity. These findings, along with the increases in single-cell migratory potential and wound healing capacity of Sur8-overexpressing cells and elevated levels of endogenous Sur8 protein expression in migrating cells, support the notion of Sur8 involvement in cell migration. Sur8 knockdown also inhibited the invasive properties of cells along with the attenuation of lung metastatic capacity of B16-F10 melanoma cells. The high Sur8 content in human metastatic melanoma tissues relative to normal skin tissues further indicates Sur8 is likely involved in tumor metastasis.

A key finding of this study is that the scaffold protein Sur8, plays an important role in mediating motility and invasive potential of cells predominantly through the PI3K pathway with a minor contribution of the ERK pathway, which agrees with previous studies showing that PI3K pathway is more important than ERK pathway in mediating metastasis [3]. Rac GTPase is a key contributor to actin cytoskeleton rearrangement and cell migration [21]. During cell invasion, mobilized cells degrade the ECM barrier to reach the bloodstream and spread to a secondary site [30]. Mechanistically, our study has found for the first time that Sur8 regulates activated Ras/PI3K pathway-induced migration and invasion of cells via activation of Rac and MMPs. The PI3K inhibition had a much greater effect on inhibiting Sur8-mediated cell motility, invasion, and activation of Rac and MMPs than did the MEK inhibition, indicating the predominant role of the PI3K pathway in these Sur8-mediated cellular behaviors. We also confirmed the regulation of PI3K pathway by Sur8 by performing p110α and Sur8 co-IP as well as by finding a direct interaction between p110α and Sur8 using purified recombinant proteins *in vitro*.

Amplification, overexpression, or mutations of epidermal growth factor receptor (EGFR) leading to the activations of both Ras-ERK and Ras-PI3K-Akt signaling pathways are detected in various human cancers [32–34]. In addition, oncogenic Ras-driven tumor malignancy is observed in one-third of all human cancers [35, 36], Ras has become a major target for the development of anti-cancer drugs. However, controlling oncogenic Ras-mediated signaling in human cancers is still difficult because most of the approaches to control oncogenic Ras, such as the identification of small Ras-binding inhibitory molecules and farnesyltransferase inhibitors that inhibit membrane localization of Ras, have been unsuccessful [37]. Moreover, the previous findings that resistance to EGFR monoclonal antibody therapies in patients with metastatic tumors is mediated by Ras mutation has further increased the importance of controlling both EGFR- and oncogenic Ras-mediated signaling [38, 39]. Thus, our identification of the role of Sur8 as a critical mediator of metastasis in human cancer through the regulation of both the PI3K-Akt and Ras-ERK pathways introduces Sur8 as a potential therapeutic target for the development of anti-cancer drugs to control tumor metastasis that arises from activated Ras and PI3K signaling.

## MATERIALS AND METHODS

### Cell culture, transfection, reagents, and drug treatment

Parental NIH3T3 cells and NIH3T3 cells containing Dox-inducible oncogenic H-Ras-overexpressing cells have been described previously [40], HEK293 and lentiviral packaging HEK293T cells were purchased from American Type Culture Collection (Manassas, VA). Human melanoma cell lines SK-5, SK-28, G361, and A375 [41] were provided by Dr. Sang Won Kang (Ewha Womans University, Seoul, Korea), and the mouse melanoma cell line B16-F10 [42] was provided by Dr. Han Woong Lee (Yonsei University, Seoul, Korea). All cells were maintained in Dulbecco's Modified Eagle Medium (DMEM; Gibco) containing 10% (v/v) fetal bovine serum (FBS; Gibco), 100 unit/ml penicillin, and 100 μg/ml streptomycin (Gibco) in 5% CO_2_ at 37°C. Cells were transfected with Lipofectamine (Invitrogen) following the manufacturer's instructions.

EGF, U0126, and LY294002 were obtained from Sigma (St. Louis). EGF was treated at a concentration of 20 ng/ml. U0126 was administered at concentrations of 20 μM for 1 hour and 10 μM for 15 hours, respectively. LY294002 was administered at concentrations of 50 μM for 1 hour and 10 μM for 15 hours, respectively. AS703026 and GDC0941 were purchased from Selleck Chemicals (Houston, TX) and were treated at concentrations of 10 μM and 1 μM, respectively. EHop-016 (Millipore) was administered at a concentration of 3 μM. Dox (Sigma) was administered at a concentration of 500 ng/ml to induce oncogenic H-Ras. Glutathione agarose beads were purchased from Sigma, and protein A/G beads were purchased from Thermo Scientific. Live cell imaging chambers were purchased from Nalgene. Matrigel-coated invasion chambers were purchased from BD Bioscience. Poly-L-ornithine (p-L-O; Sigma) and fibronectin (Gibco) were used at concentrations of 15 and 10 μg/ml, respectively. Alexa Fluor 568 phalloidin was obtained from Invitrogen.

### Plasmids, siRNAs, and shRNAs

The human Sur8 expression vector pcDNA3.1-Sur8-myc and the constructs pcDNA3.1-H-Ras-myc (H-WT-myc), pcDNA3.1-H-RasG12V-myc (H-12V-myc), pFA2-Elk-1, pFR-Luc, and pCMV-β-galactosidase (β-gal) have been described previously [43, 44]. The enhanced GFP-tagged human Sur8 (pEGFP-Sur8) was generated by cloning the full length of human Sur8 cDNA fragment into the *Bam*H1 and *Xho*1 sites of pEGFP-C1 plasmid. For the transient knockdown of Sur8 two different siRNAs siSur8#1 (5′-GCTGCGGATGCTTGATTTA-3′) and siSur8#2 (5′-AGAAATTAGTCTTGACAAA-3′) were used. The GFP siRNA (5′-GCATCAAGGTGAACTTCAA-3′) was used as a negative control. The p110α-CAAX [45] was provided by Dr. Marc B. Hershenson; and the p110α-WT [8] vector was provided by Dr. Julian Downward. The RFP-control (Con-RFP) and CA-Rac-RFP [46] plasmids were provided by Dr. Martin A. Schwartz and used for the production of DN-Rac-RFP by site directed mutagenesis using following primers: F-primer 5′ AGCTGTAGGTAAAAATTGCCTACTGATCAG 3′ and R-primer 5′ CTGATCAGTAGGCAATTTTTACC TACAGCT 3′. RFP-actin construct was provided by Dr. Naoki Watanabe, Tohoku University, Sendai, Japan. The pGL3-MMP-9-Luc and pGL3-MMP-2-Luc reporter plasmids were provided by Dr. Jung-Won Lee, Seoul National University, Seoul, Korea. The glutathione S-transferase-tagged PAK-CD (GST-PAK-CRIB Domain) plasmid [47] was provided by Dr. John G. Collard. Primers and siRNAs used were purchased from Bioneer, and all the constructs and mutations were confirmed by DNA sequencing analysis (Cosmo Genetech).

### Lentivirus production and establishment of Sur8 knockdown and overexpression cell lines

Stable knockdown of Sur8 in NIH3T3 fibroblasts was performed using a lentiviral construct described previously [44]. For the knockdown of Sur8 in melanoma cell lines we generated two different lentiviruses by inserting the target sequences shSur8#1 (5′-GCTGCGGATGCTTGATTTA-3′) and shSur8#2 (5′-AACCTTGACTTGCAGCACAAT-3′) into the pLKO.1-puromycin shRNA vector (Addgene #10878) linearized with *Age*I and *Eco*RI. For EGFP expression in this vector, EGFP-IRES was cloned in front of the puromycin-resistant marker gene driven by the *PGK* promoter using a *Bam*HI cut. For production of Dox-mediated Sur8 overexpressing lentiviral plasmid, human Sur8 cDNA was amplified by RT-PCR using F-primer 5′GCGCCGGCCGGATCCATGAGTAGTTTAGGAAAA3′ and R-primer 5′ACCACACTGGGATCCTCAGACCA TGGCACGATATGG3′ and was introduced into the pLVX-Tight-Puro vector (Clontech #632162) linearized with *Bam*HI.

Virus productions were carried out using HEK293T cells. Briefly, cells were transfected with lentiviral DNA constructs combined with the lentiviral packaging plasmids pMD2G and pAX2G in a ratio of 2:1:1, respectively. The viral supernatants were harvested 24 and 48 hours post-transfection, filtered using 0.3 μm pore filter, and used for infections. To establish stable cell lines, NIH3T3 cells were infected with either GFP-tagged Sur8 knockdown or control lentivirus and selected with puromycin to obtain single-cell clones. The single-cell clones obtained were first selected with puromycin, cultured for 2 weeks, and amplified for further experiments in culture media containing puromycin. However, B16-F10 cells were infected with a mixture of two different Sur8 knockdown lentiviruses or control lentivirus and were selected with puromycin for 2 weeks and for the further experiments cells were cultured in media containing puromycin. For conditional Sur8 overexpression, NIH3T3 cells were infected with pLVX-InSur8 lentivirus combined with a pLVX-Tet-On lentivirus (Clontech #632162) to induce Sur8 overexpression by Dox (50 ng/ml) treatment. The InConOE and InSur8OE NIH3T3 cells were selected with G418 and puromycin for 2 weeks and amplified for further experiments in culture media containing G418 and puromycin. In all the experiments, puromycin and G418 were treated at concentrations of 1 and 800 μg/ml, respectively. Primers were obtained from Bioneer, and all the constructs were confirmed by DNA sequencing analysis (Cosmo Genetech).

### Immunoblotting and IP

Immunoblottings were performed as previously described [43]. For IP, cells were lysed in IP buffer containing protease inhibitors (10 mM Tris-HCl at pH 7.5, 5 mM EDTA, 150 mM NaCl, 1% NP-40, 20 mM NaF, 1 mM sodium vanadate, 1 mM PMSF, and protease inhibitor cocktail). Whole cell lysates (WCLs) were incubated with the indicated antibodies and protein A/G agarose for 12 hours at 4°C, followed by three washes with IP buffer. Samples were boiled in 3× Laemmli sample buffer, and the immune complexes were resolved by sodium dodecyl sulfate-polyacrylamide gel electrophoresis and immunoblotted against the indicated proteins. The following antibodies were used: anti-Ras and anti-Rac1 (Upstate Biotechnology); anti-α-tubulin (Oncogene Research Products); anti-p-ERK, anti-p-Akt, anti-MEK, anti-Akt1/2, and anti-pMLC2 (Cell Signaling Technology); anti-p110α (BD Transduction Laboratories); anti-Raf-1, anti-myc, anti-Akt1, and anti-β-actin (Santa Cruz Biotechnology); and anti-GTP-Rac (NewEast Biosciences). The Sur8 polyclonal antibody was purified as described previously [44]. Horseradish peroxidase (HRP)-conjugated anti-mouse (Cell Signaling Technology) and anti-rabbit (Bio-Rad) secondary antibodies were used. Immunoblots were visualized by enhanced chemiluminescence (Amersham Bioscience) using a luminescent image analyzer (LAS-3000, Fuji Film).

### Protein purification and *in vitro* binding assay

A DNA fragment encoding the human Sur8 protein was inserted into a pGEX-4T-1 vector using *Xho*I and *Bam*HI restriction sites. Using this plasmid, recombinant human Sur8 protein was expressed in *Escherichia coli* and purified using glutathione sepharose 4B (Sigma) as previously described [48]. Recombinant His-tagged p110α (His-p110α) (Upstate Biotechnology, 3 μg) was incubated with GST or GST-Sur8 (3 μg) at 4°C for 1 hour, and then pulled down using glutathione agarose. The protein complexes were washed four times with phosphate-buffered saline (PBS; Gibco) containing 1% Tween-20. Proteins were analyzed by immunoblotting.

### Wound healing assay

The shCon-GFP and shSur8-GFP NIH3T3 and B16-F10 cells, as well as NIH3T3 cells containing Dox-inducible Sur8 or oncogenic H-Ras were seeded on p-L-O and fibronectin-coated cover glasses or chambers. After cells were grown to confluence, they were scratched with a 200 μl tip. Whenever required, EGF or the inhibitors was treated and wound healing was recorded using a time-lapse video microscope (Nikon) in a humidified 5% CO_2_ atmosphere at 37°C. Fluorescence or bright field images were captured and reconstructed into movies. The number of cells that migrated to heal the wound was quantified using the NIS-Elements AR 3.1 software (Nikon). Screenshots were captured from video files and were represented as images at several time points. For the overexpression experiments, cells were first transfected with the respective DNA. After 36 hours, cells were seeded on p-L-O and fibronectin-coated dishes and grown until confluent and scratched. In cases where real-time imaging was not performed, wound healing pictures were captured at certain time points using an ECLIPSE TE2000-U fluorescent microscope (Nikon).

### Single-cell migration assay

Stable shCon-GFP and shSur8-GFP, along with Con-GFP and Sur8-GFP overexpressing NIH3T3 cells, were seeded at a density of 1 × 10^4^ on p-L-O and fibronectin-coated chambers. After 24 hours, single cells were imaged using a time-lapse video microscope. The videos and the migratory paths of single cells were constructed using NIS-Elements AR 3.1 (Nikon). Screenshots were captured from the movie file and represented as images at several time points. For the overexpression experiments, cells were seeded on six-well plates and transfected with the respective DNA. After 36 hours, cells were seeded on p-L-O and fibronectin-coated chambers for imaging.

### Invasion assay

For invasion assays, shCon-GFP, shSur8-GFP NIH3T3 (3 × 10^4^), and B16-F10 (1 × 10^5^) cells along with NIH3T3 cells containing Dox-inducible Sur8 or oncogenic H-Ras (3 × 10^4^), were seeded on matrigel-coated chambers. In case of transient overexpression experiments, cells were first transfected with the respective DNA. Whenever required, EGF, UO126, LY294002, AS703026, GDC0941, or EHop-016 was added to the lower chambers. Cells were allowed to invade for 18 hours. After clearing the cells on the inner surface of the chamber, the cells on the outer surface were fixed using 4% paraformaldehyde (PFA) for 15 minutes and stained with crystal violet for 15 minutes. The chambers were dipped in distilled water to remove the excess staining and allowed to dry. Photographs were taken using ECLIPSE TE2000-U fluorescent microscope.

### Immunocytochemistry and phalloidin staining

Cells grown on p-L-O and fibronectin-coated cover glasses were fixed in 4% PFA for 10 minutes, followed by permeabilization with 0.1% Triton X-100 for 15 minutes, blocking in 5% bovine serum albumin (BSA) for 1 hour, and primary antibody incubation overnight at 4°C. Primary antibodies were removed, and cells were washed with PBS and incubated for 1 hour at room temperature with either Alexa Fluor 488- or Alexa Fluor 555-conjugated IgG secondary antibodies (Invitrogen). Cell nuclei were counterstained by incubating the cells in 4′, 6-diamidino-2-phenylindole (DAPI; Sigma). For cytoskeleton staining, cells were fixed with 4% PFA, permeabilized, blocked, and incubated with Alexa Fluor 568 phalloidin (1:50) for 30 minutes. Immunofluorescent images were captured using fluorescent microscope (Nikon). Confocal images were obtained by LSM 510 META microscope (Carl Zeiss).

### Rac activation assay

Rac activation assay was performed as described previously [47]. In brief, lysates of HEK293, NIH3T3, and B16-F10 cells were incubated with bacterially produced GST-PAK-CD fusion protein bound to glutathione-coupled agarose beads. The proteins bound to the fusion protein were washed three times with lysis buffer, eluted in 3× Laemmli sample buffer, and analyzed for GTP-Rac by immunoblotting with an anti-Rac1 antibody.

### Elk-1 and MMP reporter assays

For Elk-1 reporter assays, cells were transfected with a combination of the following plasmids: pFA2-Elk-1, pFR-Luc, and pCMV-β-gal. For MMP reporter assays, cells were transfected with a combination of the following plasmids: pcDNA3.1-Sur8-myc, pcDNA3.1-H-RasG12V-myc, siGFP, siSur8 #1 and #2, pGL3-MMP-9-Luc, pGL3-MMP-2-Luc, and pCMV-β-gal. Cells were harvested 36 hours post-transfection and lysed with 1× reporter lysis buffer according to the manufacturer's instructions (Promega). Whenever required, EGF treatments were 12 hours, whereas UO126 and LY294002 treatments were 15 hours. Luciferase activities were measured using FLUOstar Optima plate reader (BMG Lab Technologies) and normalized to β-gal activity as an internal control. The data represent the mean values from three independent experiments.

### RNA isolation and reverse transcription (RT)-PCR

Cells were harvested, and total RNA was isolated using TRIzol (Invitrogen). The total RNA (4 μg) was reverse transcribed in a 40 μl reaction volume using M-MLV reverse transcriptase (Invitrogen). PCR reactions were performed with *Taq*DNA polymerase (Cosmo Genetech) using the following primers synthesized by Bioneer: *Gapdh* F-primer 5′ ACCACAGTCCATGCCATCAC 3′ and R-primer 5′TCCACCACCCTGTTGCTGTA3′, *Sur8* F-primer 5′TATCCAGTGGGAGGTCCATC3′ and R-primer 5′CCTCAGGAAGGTGAGTGAGC3′, *Mmp-2* F-primer 5′GAGATCTGCAAACAGGACAT3′ and R-primer 5′GGTTCTCCAGCTTCAGGTAA3′, *Mmp-9* F-primer 5′CGACGAGTTGTGGTCGCTGG3′ and R-primer 5′GCACGCTGGAATGATCTGAG3′. PCR products were run on a 2% agarose gel, and the bands were visualized under ultraviolet illumination.

### Animals and metastasis assay

Five-week-old C57BL/6 male mice were purchased from KOATECH (Korea). Animal care and experiments were carried out according to the guidelines of the Korean Food and Drug Administration, and protocols were reviewed and approved by the Institutional Review Board of Yonsei University. Animals were fed standard rodent chow and water and maintained under a 12 hours light/12 hours dark cycle at 22–25°C with a relative humidity of 45–55%. The shCon-GFP and shSur8-GFP B16-F10 cells were trypsinized and washed in PBS. Cells (1 × 10^6^) were suspended in 150 μl PBS and injected intravenously into the tail vein of 6-week-old mice. The mice were anesthesized after 21 days, and the lungs were isolated, photographed, and fixed in 4% PFA for 3 days at 4°C. Metastasized tumor nodules on the lung surface were counted after fixation.

### Immunohistochemistry

After fixation, lung tissues were dehydrated, embedded in paraffin, and sectioned to 4 μm slices using a RM2245 microtome (Leica Microsystems). For H&E and IHC stainings, sectioned tissues were deparaffinized in xylene, hydrated in serially diluted ethanol, and stained with H&E. For IHC, the lung sections, along with the TMA containing normal human skin and malignant melanoma tissues, were autoclaved in 10 mM sodium citrate buffer (pH 6.0) for antigen retrieval and blocked in PBS containing 5% BSA and 1% goat serum at room temperature for 1 hour. The sections were incubated with anti-Sur8, -GTP-Rac, -p-Akt, or -p-ERK primary antibody overnight at 4°C. For diaminobenzidine (DAB) staining, IHC was performed using the UltraTek HRP kit (ScyTek Laboratories) and mounted in Gel/Mount media (Biomeda Corporation). All incubations were carried out in dark and humid chambers. The DAB-stained preparations were visualized using a general optical microscope (Nikon TE-2000U). For quantitative analysis, intensity of each stain was determined by HistoQuest software. In the analysis system, the cell number was first counted by checking the number and intensity of the hematoxylin stained cells. After that, DAB staining intensity of each cell was measured. Finally, hematoxylin intensity value was subtracted from DAB intensity value and the mean DAB staining was estimated.

### Statistical analysis

All statistical analyses were performed using Microsoft Excel and GraphPad Prism v.6 (GraphPad Software). All data are expressed as the mean ± standard deviation of three independent experiments. Statistical differences among the groups were analyzed by Student *t* tests and are indicated as follows: *P < 0.05; **P < 0.005; ***P < 0.0005. A *P*-value of < 0.05 was considered statistically significant.

## SUPPLEMENTAL FIGURES


