# Supplementary material for: Sur8/Shoc2 promotes cell motility and metastasis through activation of Ras-PI3K signaling
Source: Oncotarget. 2015 Sep 5;6(32):33091–105. doi: 10.18632/oncotarget.5173 (PMC4741751; doi:10.18632/oncotarget.5173)
Supplement: Supplementary file 1 [file oncotarget-06-33091-s001.pdf]

SUPPLEMENTAL FIGURES

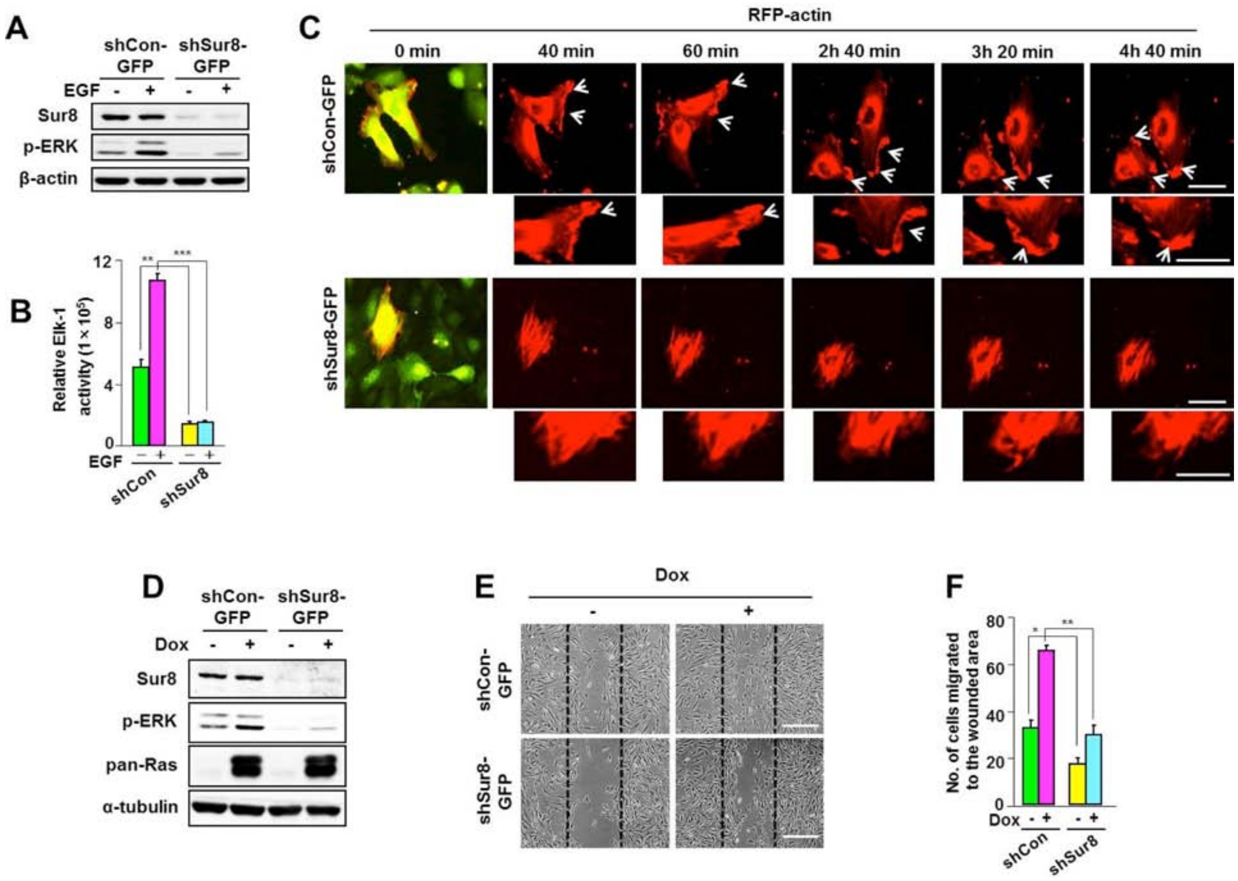

Supplementary Figure S1: Effect of Sur8 knockdown on actin cytoskeleton rearrangement, EGF- and oncogenic H-Ras-induced ERK pathway regulation and cell migration. A, B. Regulation of EGF-induced ERK pathway activation was detected by immunoblotting with an anti-p-ERK antibody (A), and by measuring Elk-1 reporter activity (B) in shCon-GFP and shSur8-GFP NIH3T3 cells. EGF was treated for 10 minutes for immunoblotting experiments and for 12 hours in reporter assay experiments. Reporter assay was performed in triplicate and values are mean ± s.e.m. **C.** Actin cytoskeleton rearrangement was analyzed in RFP-actin-expressing shCon-GFP and shSur8-GFP NIH3T3 cells using real-time imaging at indicated time points showing the actin ruffling around the cell periphery. Scale bars, 20 μm. **D–F.** Immunoblottings against indicated proteins, representative images, and quantitation in Dox-inducible oncogenic H-Ras-overexpressing NIH3T3 cells show the effects of Sur8 knockdown on oncogenic H-Ras-induced ERKs phosphorylation (D) and cell migration (E and F). Cells were treated with Dox for 24 hours before they were harvested (D) and after the cells were scratched (E) Scale bars, 200 μm. Cell migration was performed for at least three different times and the values are mean ± s.e.m.

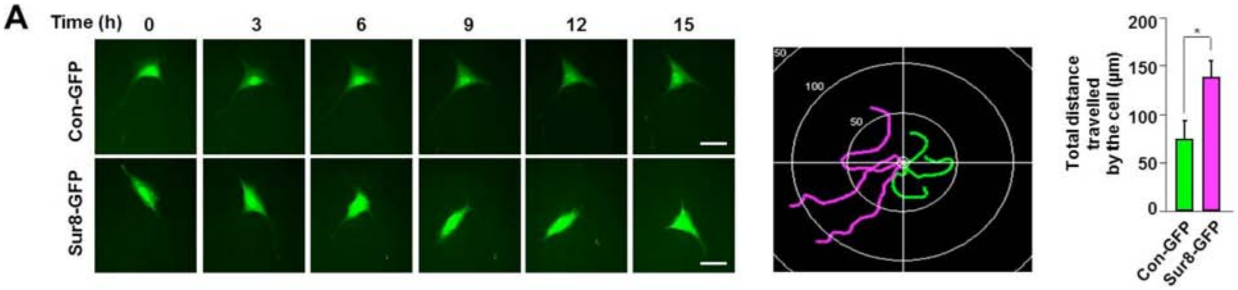

Supplementary Figure S2: Effect of Sur8 overexpression on NIH3T3 single cell migration. A. Single-cell migration abilities of NIH3T3 cells expressing Con-GFP or Sur8-GFP were recorded using real-time imaging. Screenshots were captured at the indicated time points and the distance travelled by the cells were quantified using NIS-Elements AR 3.1. Scale bars, 50 µm. The values in figures are mean ± s.e.m.

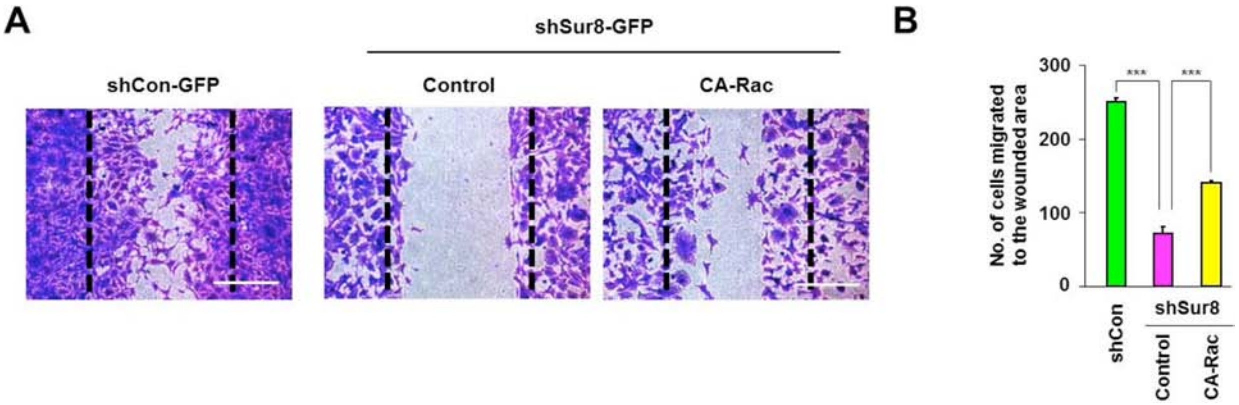

Supplementary Figure S3: Effect of CA-Rac overexpression on cell migration in Sur8 knockdown cells. A. The shCon-GFP and shSur8-GFP NIH3T3 cells were transfected with the indicated plasmids, seeded on p-L-O and fibronectin-coated plates until confluent and then scratched. After 30 hours, cells were harvested and stained with crystal violet. Representative images were captured, and the number of cells that migrated into the scratched area was quantified from three independent experiments **B.** Scale bars, 200 μm. All the values in figures are mean ± s.e.m.

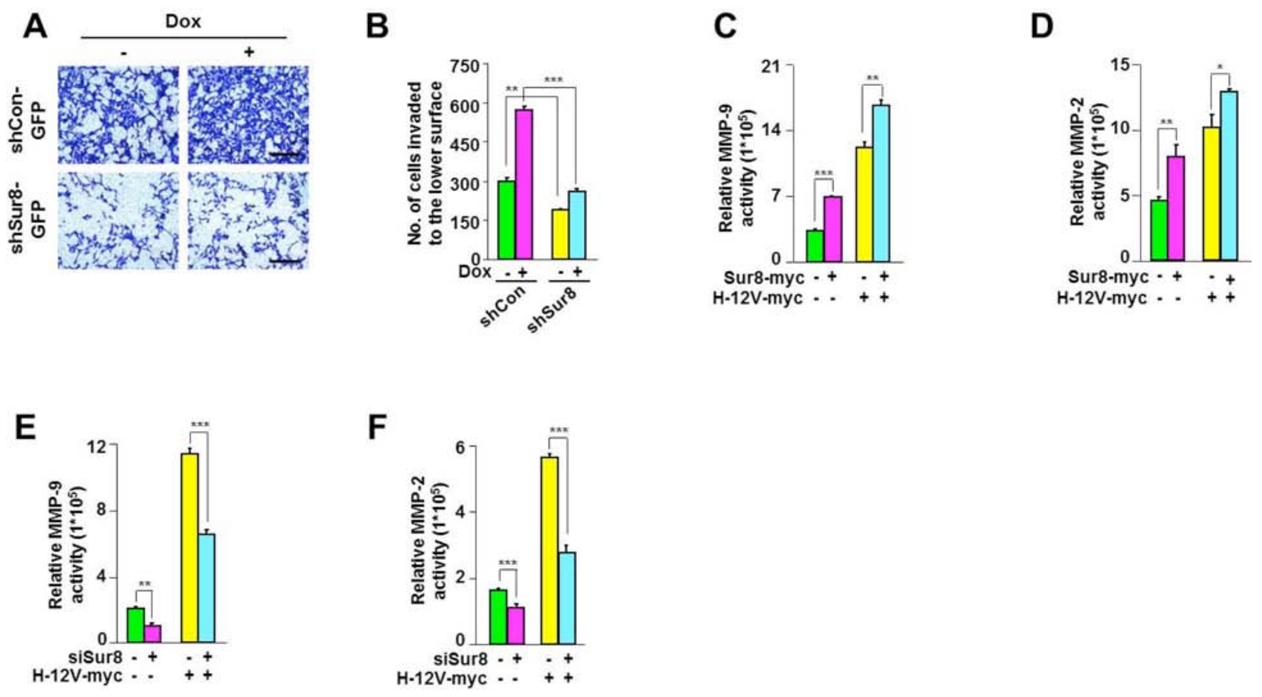

Supplementary Figure S4: Regulation of oncogenic H-Ras-induced cell invasion and MMP promoter activities by Sur8. A, B. Representative images and quantitation of cell invasion analyzed in Sur8-knockdown Dox-inducible oncogenic H-Ras-overexpressing NIH3T3 cells. Cells were seeded on matrigel-coated chambers and the cells that invaded through the matrigel were quantified. Dox was treated on the lower surface. Experiment was performed in triplicates. Scale bars, 200 μm. **C–F.** Oncogenic H-Ras-induced MMP-9 and MMP-2 promoter activities were measured in HEK293 cells with Sur8 overexpression (C, D) and knockdown (E, F) as described in Materials and methods. All the values in figures are mean ± s.e.m.

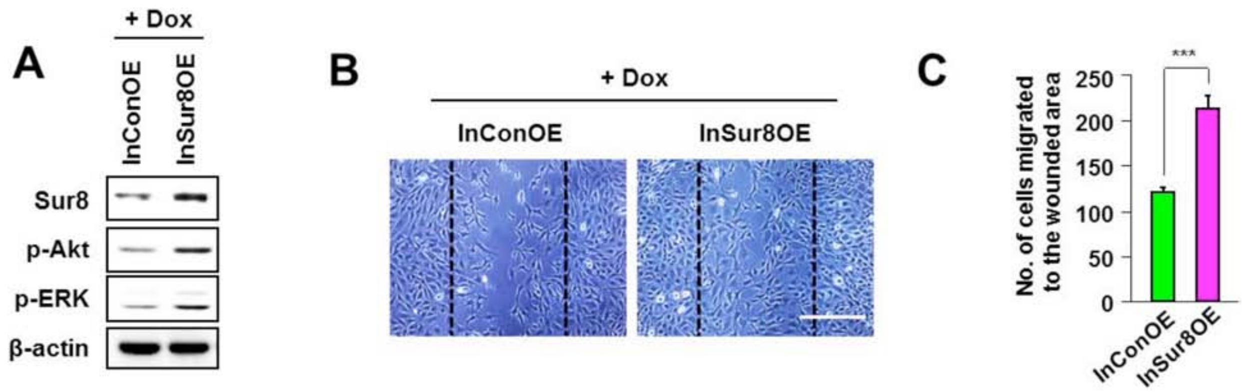

Supplementary Figure S5: Regulation of ERKs and Akt phosphorylation and cell migration by Sur8 overexpression. A–C. InConOE or InSur8OE NIH3T3 cells were used. (A) Cells were treated with Dox for 24 hours and WCLs were immunoblotted with antibodies against the indicated proteins. (B) Cells were treated with Dox for 72 hours, seeded, and scratched when they were confluent. Bright field images of the cells were captured. The number of cells that migrated to heal the wound was counted from three independent experiments (C) Scale bar, 200 µm.

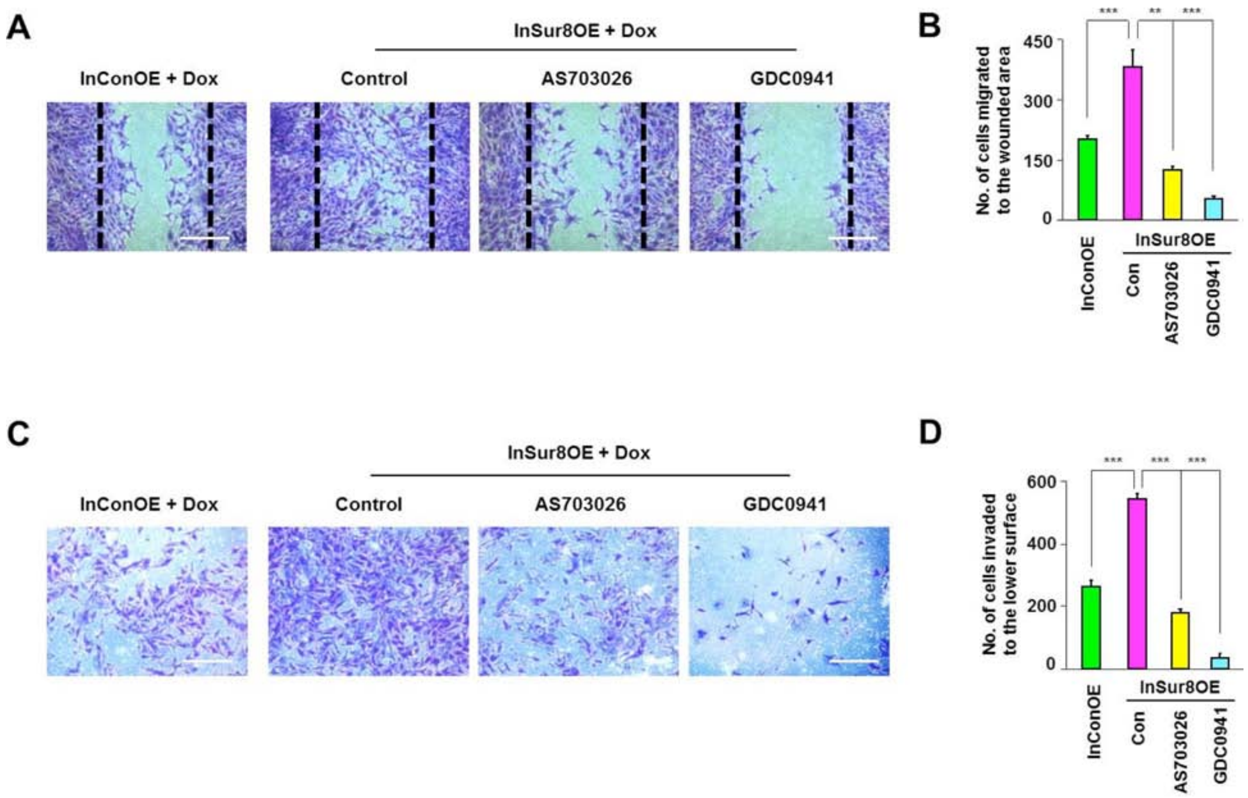

Supplementary Figure S6: Effects of AS703026 and GDC0941 on Sur8-mediated migration and invasion of cells. A–D. InConOE or InSur8OE NIH3T3 cells were used. (A) Cells were seeded, grown until confluent, and scratched. Cells were either treated or untreated with AS703026 or GDC0941. After 30 hours, cells were stained with crystal violet and the number of cells that migrated to the scratched area was counted from three independent experiments (B) Scale bars, 200 μm. (C) Cells were seeded on matrigel-coated chambers and either treated or untreated with AS703026 or GDC0941 on the lower surface. Cells that invaded through the matrigel were stained with crystal violet. Representative images were captured, and invaded cells were counted from three independent experiments (D) Scale bars, 200 μm. Cells in figures A–D were treated with Dox for 72 hours to induce Sur8 overexpression. All the values in figures are mean ± s.e.m.

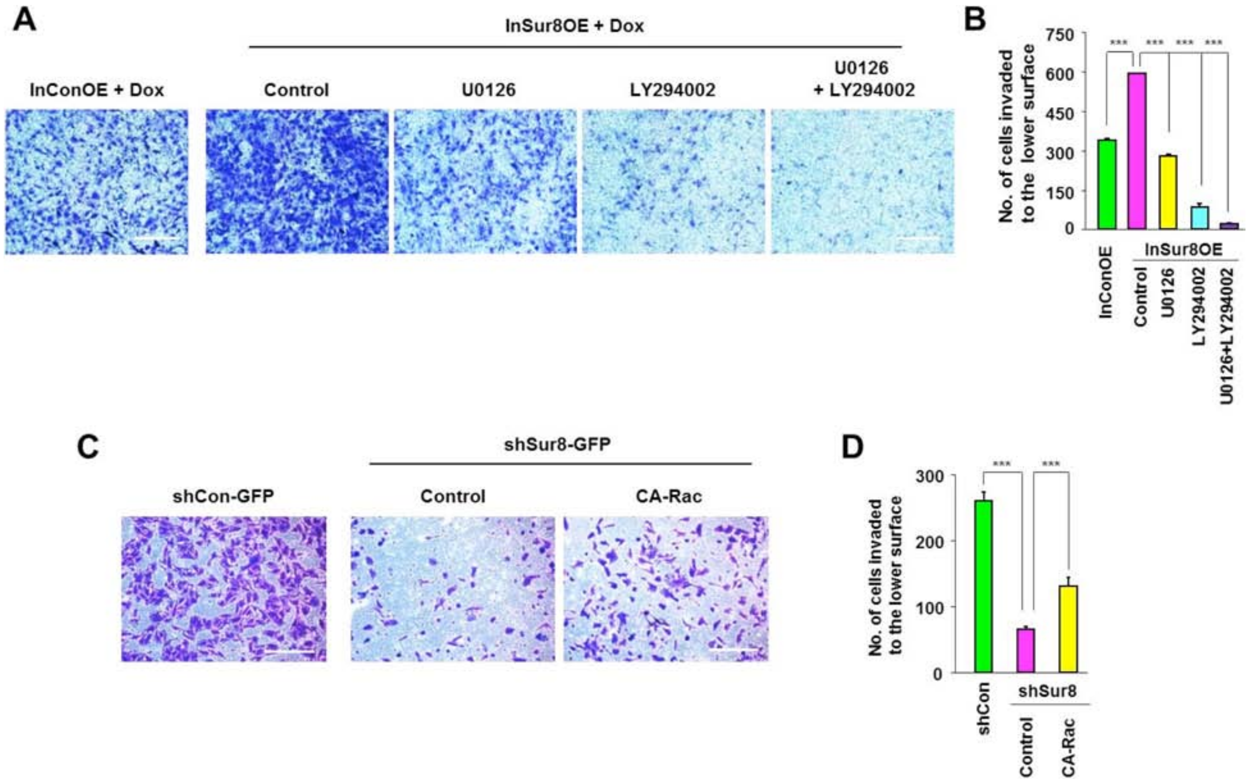

Supplementary Figure S7: The effects of U0126 and/or LY294002 on Sur8-mediated invasion (A, B), and CA-Rac overexpression on cell invasion in Sur8 knockdown cells (C, D). A. The InConOE and InSur8OE cells were treated with Dox for 72 hours and seeded on matrigel-coated chambers that were treated or non-treated with U0126 and/or LY294002 on the lower surface. Cells that invaded through the matrigel were stained with crystal violet, representative images were captured, and the invaded cells were counted from three independent experiments **B.** Scale bars, 200 µm. **C.** The shCon-GFP or shSur8-GFP NIH3T3 cells were transfected with the indicated plasmids and seeded on matrigel-coated chambers. Cells that invaded through the matrigel were stained with crystal violet. Representative images were captured, and the total number of invaded cells was quantified from three independent experiments **D.** Scale bars, 200 µm. All the values in figures are mean ± s.e.m.

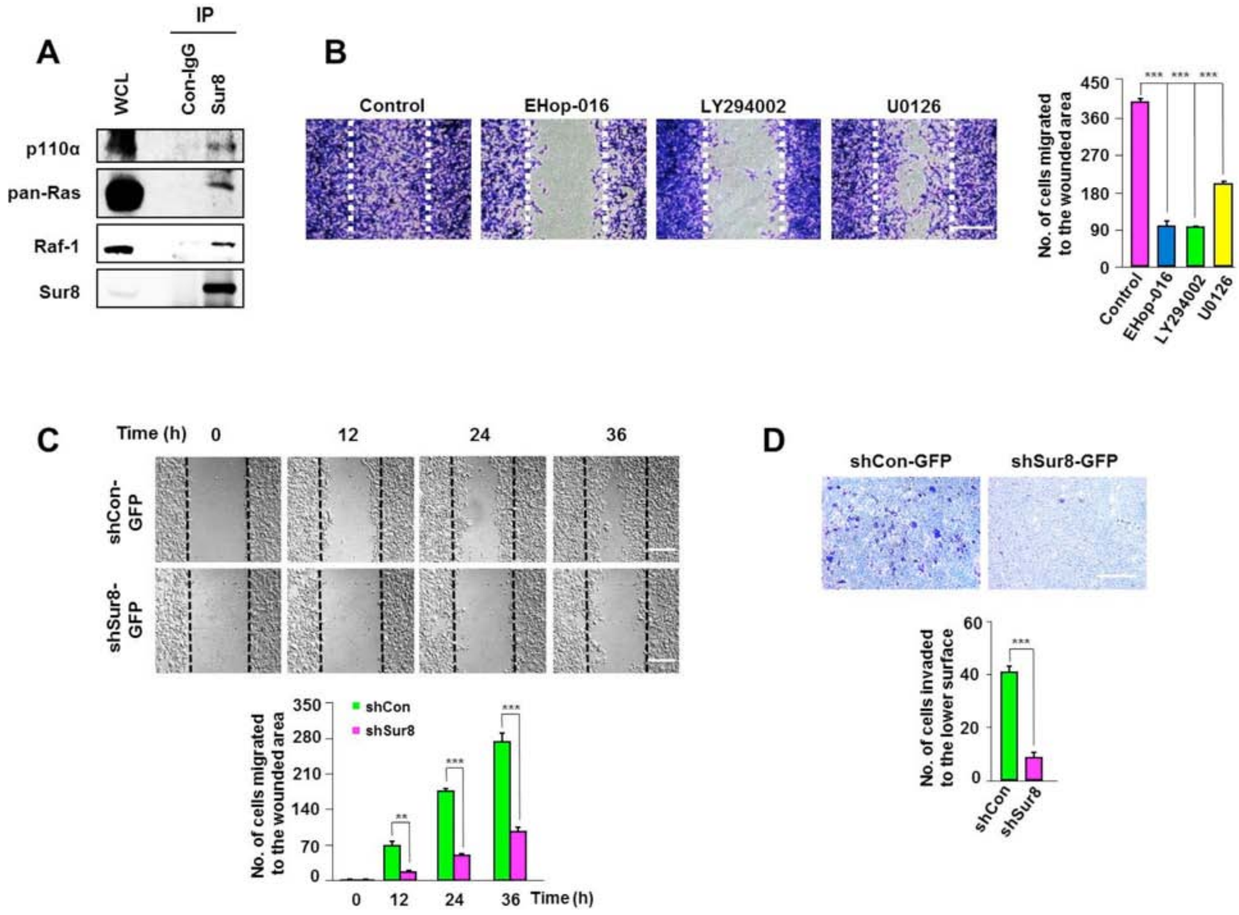

Supplementary Figure S8: Interaction of Sur8 with p110α, Ras, and Raf-1 (A) and the effects of inhibition of ERK, PI3K, or Rac activity (B), and Sur8 knockdown on cell migration and invasion (C, D) in B16-F10 cells. A. WCLs of B16-F10 cells were immunoprecipitated with either control IgG or Sur8 antibody and immunoblotting was performed against the indicated proteins. **B.** Confluent B16-F10 cells were scratched and treated with U0126, LY294002, or EHop-016 for 24 hours and stained with crystal violet. Representative images were captured, and the number of cells that migrated into the scratched area were quantified. Three independent experiments were performed. Scale bar, 200 μm. **C, D.** shCon-GFP and shSur8-GFP B16-F10 cells were used. (C) Cells were grown to confluence and scratched, bright field images were captured at different time points. The number of cells that migrated into the wounded area were counted. Three independent experiments were performed. Scale bars, 250 μm. (D) Cells were seeded on matrigel-coated chambers, and those that invaded through the matrigel were stained with crystal violet. Representative images were captured, and the total number of invaded cells is presented. Three independent experiments were performed. Scale bar, 200 μm. All the values in figures are mean ± s.e.m.
